# Supplementary material for: Knowledge and Attitudes of Guam Residents towards Cancer Clinical Trial Participation
Source: Int J Environ Res Public Health. 2022 Nov 29;19(23):15917. doi: 10.3390/ijerph192315917 (PMC9736365; doi:10.3390/ijerph192315917)
Supplement: Supplementary file 1 [file ijerph-19-15917-s001.zip › 20221128-Taafaki Manuscript. Supp Table S1. KA Survey Questions.pdf]

Supplementary Table S1: Knowledge and Attitute Survey Questions

Type	Question
Knowledge 1	Have you heard of the term 'clinical trial'?
Knowledge 2	Do you agree or disagree with the following statement? "Clinical trials test how safe and useful a new drug is against cancer and other diseases".
Knowledge 3	Does taking part in a clinical trial mean you might not receive the treatment being tested?
Knowledge 4	Do you agree or disagree with the following statement? In a clinical trial, the sponsor pays for the new drug being tested while all other costs are billed to your insurance company.
Attitude 5	Have you ever participated in a clinical trial?
Attitude 6	Has anyone you know taken part in a clinical trial?
Attitude 7	If you had cancer and were asked to be in a cancer clinical trial, would you take part?
Attitude 8	If you had cancer and were offered a cancer clinical trial, would you take part if it meant you needed to leave Guam for treatment?
Attitude 9	If you had cancer, would you prefer to take part in a cancer clinical trial offered in Guam rather than going off island for the same treatment?
Attitude 10	If you had cancer, do you believe you would benefit by taking part in a clinical trial in Guam?
Attitude 11	If you had cancer, would you take part in a clinical trial if you believed it would help other people in your community?
Attitude 12	If you had cancer, would you take part in a clinical trial if you believed it would lead to new treatments for cancer?
Attitude 13	Do you think you would receive good quality treatment from a clinical trial offered in Guam?
Attitude 14	Do you think that people who take part in cancer clinical trials are treated like 'guinea pigs'?
Attitude 15	Would the possibility of serious side effects stop you from taking part in a clinical trial?
Attitude 16	Do you think your health insurance would cover you if you took part in a clinical trial in Guam?
Attitude 17	Do you think you would have to pay more out-of-pocket expenses if you took part in a clinical trial in Guam?
Attitude 18	If you had cancer, would you want your doctor to offer you a clinical trial in Guam?
Attitude 19	If you had cancer, would you be willing to change doctors in order to take part in a clinical trial in Guam?
Attitude 20	If you had cancer, would the doctor's ethnicity be important in your decision to take part in a cancer clinical trial?
Attitude 21	Would you be willing to take part in a cancer clinical trial if the doctor was not your ethnicity?
Attitude 22	If your doctor gave you advice that goes against your cultural beliefs, would you listen to them?
Attitude 23	Do you trust western medicine?
Attitude 24	How important is having the emotional support of your family or social group in your decision to take part in a cancer clinical trial?
Attitude 25	How important is the support of your religious community if you decided to take part in a cancer clinical trial?
Attitude 26	If you had cancer, would you seek traditional healing practices?
Attitude 27	Have you ever gone to a *suruhano* for health care?
Attitude 28	Can a suruhano treat cancer?
Attitude 29	If you had cancer, would you seek treatment by a *suruhano*?
Attitude 30	Do you believe cancer is caused by *taotaomo'na* (ancient spirits)?
Attitude 31	Do you trust traditional medicine?
