# Supplementary material for: Knowledge and Attitudes of Guam Residents towards Cancer Clinical Trial Participation
Source: Int J Environ Res Public Health. 2022 Nov 29;19(23):15917. doi: 10.3390/ijerph192315917 (PMC9736365; doi:10.3390/ijerph192315917)
Supplement: Supplementary file 1 [file ijerph-19-15917-s001.zip › 20221128-Taafaki Manuscript. Supp. Table S2. Univariate Logistics Regression cOR-1.pdf]

**Supplementary Table S2.** Crude Odds Ratio and 95% Confidence Interval of Univariate Logistic Regressions

| Variable | Knowledge 1: Have you heard of the term 'clinical trial'? | Knowledge 3. Does taking part in a clinical trial mean you might not receive the treatment being tested? | Knowledge 4. In a clinical trial, the sponsor pays for the new drug being tested while all other costs are billed to your insurance company | Attitude 13. Do you think you would receive good quality treatment from a clinical trial offered in Guam? | Attitude 14. Do you think that people who take part in cancer clinical trials are treated like 'guinea pigs? | Attitude 17. Do you think you would have to pay more out-of-pocket expenses if you took part in a clinical trial in Guam? | Attitude 20. If you had cancer, would the doctor's ethnicity be important in you decision to take part in a cancer clinical trial? | Attitude 22. If your doctor gave you advice that goes against your cultural beliefs, would you listen to them? | Attitude 25. How important is the support of your religious community if you decided to take part in a cancer clinical trial? | Attitude 26. If you had cancer, would you seek traditional healing practices? | Attitude 27. Have you ever gone to a *suruhano* for health care? | Attitude 29. If you had cancer, would you seek treatment by a *suruhano*? |
|---|---|---|---|---|---|---|---|---|---|---|---|---|
| Age | 0.99 (0.96-1.01) | 0.98 (0.95-1.00) | 1.00 (0.98-1.03) | 1.02 (0.99-1.04) | 0.99 (0.97-1.02) | 0.99 (0.97-1.02) | 1.02 (0.99-1.04) | 1.01 (0.98-1.03) | 1.00 (0.98-1.03) | 0.99 (0.97-1.02) | 1.02 (0.99-1.04) | 0.97 (0.95-0.99)* |
| Gender (Ref: Male) | | | | | | | | | | | | |
| *Female* | 1.32 (0.69-2.53) | 0.83 (0.41-1.66) | 0.82 (0.42-1.62) | 1.32 (0.69-2.53) | 0.97 (0.50-1.90) | 1.49 (0.78-2.84) | 0.69 (0.34-1.37) | 0.55 (0.28-1.08) | 0.83 (0.44-1.57) | 1.75 (0.88-3.46) | 1.73 (0.90-3.34) | 1.01 (0.52-1.95) |
| Ethnicity (Ref: White) | | | | | | | | | | | | |
| *Chamorro* | 0.18 (0.04-0.86)* | 0.45 (0.12-1.71) | 0.88 (0.27-2.82) | 0.72 (0.23-2.28) | 1.96 (0.51-7.54) | 5.34 (1.68-17.00)** | 300000.00 (0.00-****) | 0.64 (0.20-2.01) | 27.70 (3.47-221.00)** | 1.14 (0.37-3.46) | 10.20 (2.18-48.20)** | 2.08 (0.66-6.53) |
| *Filipino* | 0.09 (0.02-0.44)** | 0.36 (0.09-1.45) | 2.17 (0.65-7.31) | 0.56 (0.17-1.90) | 4.22 (1.05-17.00)* | 2.40 (0.71-8.07) | 500000.00 (0.00-****) | 0.63 (0.18-2.12) | 42.20 (4.98-357.0)** | 1.13 (0.34-3.74) | 3.61 (0.72-18.20) | 0.80 (0.23-2.83) |
| *Other* | 0.38 (0.07-2.16) | 1.02 (0.20-5.29) | 1.05 (0.27-4.13) | 0.32 (0.08-1.21) | 3.59 (0.81-16.00) | 2.20 (0.58-8.28) | 290000.00 (0.00-****) | 1.18 (0.29-4.78) | 8.53 (0.95-76.60) | 1.02 (0.27-3.80) | 2.65 (0.46-15.10) | 1.54 (0.40-5.88) |
| Marital Status (Ref: Married/Living as Married) | | | | | | | | | | | | |
| *Single* | 0.75 (0.35-1.60) | 0.79 (0.35-1.81) | 0.87 (0.39-1.96) | 0.41 (0.19-0.89)* | 1.00 (0.46-2.19) | 1.64 (0.76-3.54) | 1.02 (0.45-2.29) | 0.80 (0.37-1.73) | 1.34 (0.63-2.89) | 0.56 (0.25-1.25) | 0.89 (0.41-1.91) | 0.74 (0.34-1.63) |
| *Divorced/widowed/separated* | 0.88 (0.32-2.41) | 0.39 (0.14-1.08) | 1.15 (0.41-3.21) | 1.16 (0.40-3.33) | 0.64 (0.21-1.93) | 1.26 (0.46-3.42) | 1.06 (0.36-3.06) | 1.21 (0.42-3.49) | 0.61 (0.22-1.66) | 0.32 (0.12-0.88)* | 1.03 (0.38-2.81) | 0.87 (0.31-2.41) |
| Education (Ref: ≤High school graduate) | | | | | | | | | | | | |
| *Some college or technical school* | 2.14 (0.79-5.80) | 0.94 (0.35-2.58) | 0.93 (0.35-2.49) | 1.67 (0.61-4.61) | 0.72 (0.25-2.06) | 0.96 (0.34-2.70) | 0.31 (0.10-0.96)* | 3.42 (1.17-10.00)* | 0.94 (0.35-2.58) | 0.79 (0.29-2.13) | 0.70 (0.25-1.92) | 0.27 (0.08-0.92)* |
| *≥College graduate* | 5.04 (2.34-10.90)*** | 2.34 (1.06-5.19)* | 0.34 (0.16-0.74)** | 1.52 (0.74-3.12) | 0.91 (0.44-1.90) | 0.45 (0.22-0.93)* | 0.31 (0.15-0.68)** | 3.20 (1.52-6.75)** | 0.56 (0.27-1.15) | 1.72 (0.80-3.69) | 0.96 (0.47-1.97) | 0.91 (0.44-1.86) |
| Personal Income (Ref: <$50,000) | | | | | | | | | | | | |
| *$50,000 or more* | 2.44 (1.18-5.02)* | 1.79 (0.83-3.84) | 0.77 (0.37-1.62) | 2.44 (1.18-5.02)* | 0.60 (0.29-1.22) | 0.52 (0.26-1.03) | 0.34 (0.16-0.75)** | 1.67 (0.82-3.40) | 0.65 (0.33-1.28) | 1.35 (0.65-2.80) | 1.40 (0.70-2.80) | 1.57 (0.78-3.18) |
| *Refused* | 0.50 (0.17-1.50) | 0.74 (0.25-2.19) | 2.30 (0.79-6.70) | 0.82 (0.28-2.36) | 0.29 (0.08-1.10) | 0.84 (0.29-2.46) | 0.60 (0.19-1.87) | 1.35 (0.45-4.06) | 2.40 (0.71-8.07) | 1.01 (0.34-3.06) | 1.19 (0.41-3.50) | 2.45 (0.84-7.15) |
| Born Country (Ref: USA) | | | | | | | | | | | | |
| *Guam* | 0.50 (0.18-1.40) | 0.89 (0.33-2.41) | 1.13 (0.41-3.06) | 0.67 (0.26-1.73) | 1.86 (0.62-5.54) | 2.84 (1.13-7.14)* | 2.28 (0.70-7.40) | 0.60 (0.23-1.54) | 5.29 (1.90-14.70)** | 1.00 (0.38-2.64) | 7.80 (2.45-24.90)** | 1.13 (0.45-2.81) |
| *Philippines* | 0.18 (0.06-0.57)** | 0.81 (0.26-2.55) | 2.39 (0.79-7.27) | 0.57 (0.19-1.69) | 6.14 (1.84-20.50)** | 1.81 (0.63-5.19) | 3.76 (1.05-13.50)* | 0.74 (0.25-2.22) | 7.33 (2.25-23.90)** | 0.65 (0.22-1.93) | 2.15 (0.58-8.01) | 0.45 (0.14-1.42) |
| *Other* | 0.50 (0.13-1.95) | 1.11 (0.27-4.60) | 1.23 (0.31-4.84) | 0.57 (0.16-2.08) | 2.52 (0.62-10.30) | 2.06 (0.58-7.29) | 3.30 (0.76-14.30) | 1.93 (0.43-8.69) | 2.59 (0.68-9.95) | 0.98 (0.25-3.76) | 1.83 (0.39-8.67) | 1.60 (0.45-5.63) |
| Employment Status (Ref: Employed) | | | | | | | | | | | | |
| *Retired* | 0.70 (0.29-1.71) | 0.37 (0.15-0.93)* | 1.46 (0.58-3.70) | 1.60 (0.63-4.04) | 1.85 (0.77-4.45) | 1.20 (0.50-2.88) | 0.96 (0.36-2.56) | 0.89 (0.35-2.22) | 1.07 (0.45-2.56) | 0.52 (0.21-1.28) | 0.92 (0.38-2.25) | 0.63 (0.25-1.59) |
| *Unemployed/student/homemaker/unable to work* | 0.34 (0.15-0.80)* | 0.35 (0.15-0.85)* | 3.15 (1.35-7.38)** | 0.93 (0.40-2.13) | 0.56 (0.22-1.45) | 1.52 (0.65-3.54) | 2.00 (0.85-4.69) | 0.41 (0.18-0.95)* | 1.59 (0.68-3.70) | 0.57 (0.24-1.35) | 1.29 (0.56-2.95) | 0.71 (0.30-1.67) |
| English Fluency (Ref: Not well): | | | | | | | | | | | | |
| *Well* | 9.57 (2.04-44.90)** | 1.08 (0.31-3.70) | 0.07 (0.02-0.34)** | 1.32 (0.42-4.13) | 0.61 (0.19-1.92) | 3.16 (0.93-10.80) | 0.24 (0.07-0.78)* | 1.09 (0.34-3.50) | NA | 0.35 (0.07-1.64) | 0.81 (0.26-2.53) | 2.17 (0.57-8.24) |
| *Speaking Other Language* | 0.27 (0.12-0.61)** | 0.87 (0.40-1.90) | 2.45 (1.07-5.63)* | 1.06 (0.52-2.16) | 1.97 (0.90-4.32) | 1.41 (0.70-2.85) | 3.84 (1.49-9.89)** | 0.47 (0.21-1.03) | 2.52 (1.22-5.18)* | 0.82 (0.38-1.75) | 1.77 (0.85-3.72) | 1.10 (0.53-2.29) |
| Insurance (Ref: Private) | | | | | | | | | | | | |
| *Public (Medicare, Medicaid, military)* | 0.45 (0.21-0.98)* | 0.59 (0.26-1.36) | 5.23 (2.27-12.0)*** | 0.69 (0.32-1.49) | 0.76 (0.34-1.70) | 1.18 (0.55-2.51) | 1.78 (0.78-4.03) | 0.56 (0.26-1.21) | 1.95 (0.90-4.22) | 1.01 (0.45-2.29) | 1.03 (0.49-2.20) | 0.75 (0.34-1.63) |
| *No insurance* | 0.24 (0.08-0.68)** | 0.26 (0.09-0.72)* | 6.51 (2.24-18.9)** | 0.57 (0.21-1.54) | 1.60 (0.59-4.35) | 0.97 (0.36-2.61) | 3.67 (1.31-10.30)* | 1.28 (0.42-3.92) | 1.44 (0.53-3.93) | 0.48 (0.18-1.32) | 0.24 (0.06-0.87)* | 0.50 (0.16-1.50) |
| Have Religion | 0.47 (0.19-1.15) | 0.69 (0.27-1.74) | 0.92 (0.39-2.15) | 1.51 (0.67-3.41) | 1.02 (0.44-2.39) | 1.46 (0.65-3.29) | 1.51 (0.60-3.84) | 0.38 (0.14-1.00) | NA | 1.32 (0.57-3.05) | 5.57 (1.83-17.00)** | 1.01 (0.44-2.33) |

NA = Non available due to zero in a cell. *P<0.05. **P<0.01. ***P<0.001.
